# Myosteatosis as a prognostic factor of *Mycobacterium avium* complex pulmonary disease

**DOI:** 10.1038/s41598-023-40984-y

**Published:** 2023-08-22

**Authors:** Eunki Chung, Youngmok Park, Song Yee Kim, Moo Suk Park, Young Sam Kim, Hye-Jeong Lee, Young Ae Kang

**Affiliations:** 1 Division of Pulmonary and Critical Care Medicine, Department of Internal Medicine, Severance Hospital, Institute for Immunology and Immunological Disease, Yonsei University College of Medicine, Seoul, Republic of Korea; 2 Department of Radiology, Research Institute of Radiological Science, Severance Hospital, Yonsei University College of Medicine, 50-1 Yonsei-ro, Seodaemun-gu, Seoul, 03722 Republic of Korea

**Keywords:** Respiratory tract diseases, Infectious diseases, Bacterial infection, Muscle

## Abstract

Quantitative body composition affects the prognosis of patients with *Mycobacterium avium* complex pulmonary disease (MAC-PD). However, whether the qualitative body composition obtained indirectly through computed tomography (CT) affects their prognosis is debatable. We retrospectively analyzed patients with MAC-PD who underwent non-contrast CT at MAC-PD diagnosis. The cross-sectional area of the erector spinae muscle (ESM area), the Hounsfield unit of the erector spinae muscle (ESM HU), and the cross-sectional area of subcutaneous fat (SQF area) were measured at the level of the first lumbar vertebra. Myosteatosis were defined below the median value of ESM HU for each sex. Of 377 patients, 45 (11.9%) died during the follow-up. Patients who died were older and had a lower ratio of females (33.3%). In body compositions, SQF area and ESM HU were lower in the patients who died. In multivariable analysis, a low ESM HU was associated with increased mortality (ESM HU adjusted hazard ratio [aHR] 0.95, 95% confidence interval [CI] 0.93–0.97) through body composition. SQF area revealed protective effects in MAC-PD patients with body mass index ≥ 18.5 kg/m^2^ (aHR 0.98, 95% CI 0.95–1.00). In conclusion, the decrease in ESM HU, which indirectly reflects myosteatosis, is associated with mortality in patients with MAC-PD.

## Introduction

The incidence of *Mycobacterium avium* complex (MAC) pulmonary disease (MAC-PD), accounting for a large proportion of nontuberculous mycobacteria (NTM) pulmonary disease (NTM-PD), is continuously increasing^1,2^. As NTM-PD has high unsuccessful treatment outcome^3^ and mortality^4^ rates, it is essential to reduce modifiable risk factors in addition to medical treatment. Among the widely known modifiable risk factors^5^, low body mass index (BMI) affects NTM-PD development^6^ and mortality^7^. However, even with the same BMI, there can be variations in the ratio of muscle to fat. It's established that both muscle and fat exert independent impacts on the development^8^ and prognosis of NTM-PD^9^. Therefore, evaluating the influence of each patient's body composition becomes imperative. Furthermore, considering reduced survival rates among cancer patients and cirrhosis patients based on the distribution of fat, even with the same total body fat mass and percentage, it might be worth considering fat distribution in NTM-PD patients as well^10,11^.

Aging or inflammation-induced lipolysis can induce ectopic fat infiltration, leading to the presence of fat within the skeletal muscle. This condition is referred to as myosteatosis^12^. Through the utilization of computed tomography (CT) scans in NTM-PD diagnosis, it is possible to differentiate between muscle and fat^13^, enabling quantitative measurements^14^. Furthermore, muscle attenuation measured in Hounsfield units provides a non-invasive means to assess myosteatosis^15^. In two previous studies, the cross-sectional area of the erector spinae muscle (ESM area) and Hounsfield unit (HU) of the erector spinae muscle (ESM HU) were measured with CT to predict MAC-PD prognosis based on body composition^9,16^. However, as the results for ESM HUs are inconsistent, further research is necessary.

Thus, this study aimed to investigate the prognostic significance of myosteatosis and quantitative body composition measured at the first lumbar vertebra (L1) level using chest CT regarding the survival of patients with MAC-PD.

## Results

**Figure 1:**
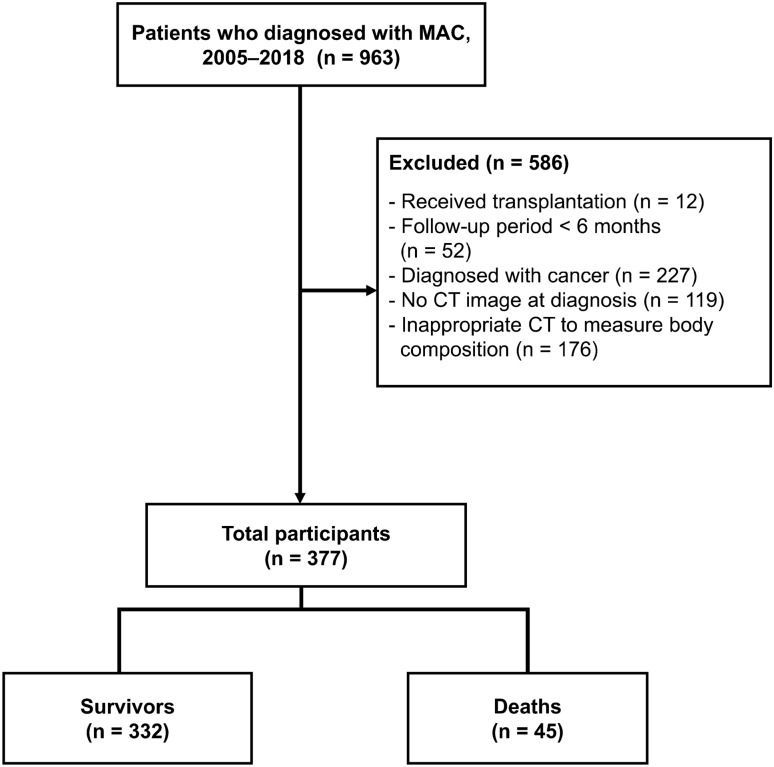
Flow chart of participants with *Mycobacterium avium* complex pulmonary disease (MAC-PD). MAC, *Mycobacterium avium* complex; CT, computed tomography.

A retrospective cohort of 963 patients diagnosed with MAC-PD between 2005 and 2018 at the tertiary referral hospital was eligible for analysis. After excluding 586 people (received transplantation, follow-up period < 6 months, diagnosed with cancer, no CT image at diagnosis, inappropriate CT for measure body composition), 377 participants were included in the final analysis (Fig. 1).

### Baseline characteristics

Table 1 presents baseline characteristics of patients with MAC-PD in the entire group and subgroups according to sex. The females were younger (61.7 years vs. 68.6 years) and had better nutritional status represented by the prognostic nutritional index (PNI)^17^ than males. Acid-fast bacilli (AFB) positivity, CT scores, treatment times, and BMI did not statistically differ between males and females. However, individual body compositions differed. In females, the cross-sectional area of subcutaneous fat (SQF area) was > 70% higher than that in males based on the median value. In contrast, ESM area and ESM HU were low; therefore, the fat composition ratio was higher in females than that in males.

**Table 1:** Baseline characteristics of participants with *Mycobacterium avium* complex pulmonary disease (MAC-PD) according to sex.

Variable	Total participants (n = 377)	Males (n = 120)	Females (n = 257)	*p*-value
Age (years)*	63.88 ± 11.40	68.60 ± 10.06	61.68 ± 11.34	< 0.001
Smoking status (Current or former), no. (%)	66 (17.5)	63 (52.5)	3 (1.2)	< 0.001
Height, cm*	160.10 ± 7.73	167.40 ± 5.84	156.64 ± 5.88	< 0.001
Weight, kg**	53.00 (47.00–60.00)	60.00 (50.50–65.00)	52.00 (46.00–56.00)	< 0.001
BMI, kg/m^2^*^a^	20.80 ± 3.02	20.93 ± 3.21	20.74 ± 2.93	0.583
Comorbidities, no. (%)				
History of TB	155 (41.1)	59 (49.2)	96 (37.4)	0.039
COPD	33 (8.8)	22 (18.3)	11 (4.3)	< 0.001
Asthma	29 (7.7)	7 (5.8)	22 (8.6)	0.473
DM	41 (10.9)	17 (14.2)	24 (9.3)	0.221
Cardiovascular disease	44 (11.7)	29 (24.2)	15 (5.8)	< 0.001
Liver disease	20 (5.3)	9 (7.5)	11 (4.3)	0.293
Sputum smear positivity, no. (%)	175 (46.4)	59 (49.2)	116 (45.1)	0.535
CT pattern, no. (%)				0.004
FC	42 (11.1)	21 (17.5)	21 (8.2)	
NB with cavity	58 (15.4)	11 (9.2)	47 (18.3)	
NB without cavity	277 (73.5)	88 (73.3)	189 (73.5)	
CT score**	8.00 (6.00–10.00)	8.00 (6.00–10.00)	8.00 (6.00–10.00)	0.363
Cavity, no. (%)				1.000
No cavity	277 (73.5)	88 (73.3)	189 (73.5)	
Cavity	100 (26.5)	32 (26.7)	68 (26.5)	
PNI**	50.95 (47.48–53.50)	48.80 (44.90–51.45)	51.53 (48.39–54.55)	< 0.001
Follow-up duration (days)**	1638.00 (838.00–2587.00)	1719.50 (749.25–2666.50)	1567.00 (851.00–2570.50)	0.937
Treatment times, no. (%)				0.175
None	199 (52.8)	58 (48.3)	141 (54.9)	
Once	155 (41.1)	57 (47.5)	98 (38.1)	
Twice	23 (6.1)	5 (4.2)	18 (7.0)	
SQF area**	52.98 (34.18–77.69)	36.79 (21.13–53.77)	63.94 (41.45–86.30)	< 0.001
ESM area**	26.93 (22.97–31.42)	32.54 (27.03–36.19)	25.36 (21.64–28.89)	< 0.001
ESM HU**	36.98 (30.57–43.17)	40.54 (33.42–46.34)	36.06 (28.72–41.36)	< 0.001

*Mean ± standard deviation.

**Median (IQR).

^a^Seven people without BMI data were excluded from the participants.

BMI, body mass index; TB, tuberculosis; COPD, chronic obstructive pulmonary disease; DM, diabetes mellitus; CT, computed tomography; FC, fibrocavitary; NB, nodular bronchiectatic; PNI, prognostic nutrition index; SQF area, L1 spine level of the cross-sectional area of subcutaneous fat; ESM area, L1 spine level of the cross-sectional area of the erector spinae muscle; ESM HU, L1 spine level of Hounsfield unit of the erector spinae muscle; IQR, interquartile range.

The different characteristics of survivors and deceased patients with MAC-PD are presented in Table 2. Of 377 patients with MAC-PD (mean age, 63.9 years), 45 (11.9%) died during the 4.5-year follow-up. Among the deceased patients, respiratory diseases (n = 18) were the primary cause of death, while other causes of death included sepsis (n = 5), cardiovascular diseases (n = 4), cerebrovascular diseases (n = 3), Alzheimer’s disease (n = 1), kidney diseases (n = 1), connective tissue diseases (n = 2), hematologic diseases (n = 2), and other diseases (n = 9). The group that died was older (74.1 years vs. 62.5 years) and had a higher percentage of males (66.7% vs. 27.1%) than the survivor group. Patients with MAC-PD in the group that died had a lower BMI, more underlying diseases, poor CT scores, more cavities measured using CT, and poor nutritional status than survivors. However, no differences were observed between the death and survivor groups in AFB positivity and treatment times. In body compositions, SQF area and ESM HU were lower in the group that died. However, the ESM area did not differ between both groups. In subgroup analysis, the differences in all the CT-measured body compositions were significant in females, but in males, SQF area and ESM area were significantly different between the survivor and deceased patient groups (Supplementary Tables S1–S2).

**Table 2:** Different characteristics of survivors and deceased patients with MAC-PD.

Variable	Total participants (n = 377)	Survivors (n = 332)	Deaths (n = 45)	*p*-value
Age (years)*	63.88 ± 11.40	62.50 ± 10.95	74.07 ± 9.45	< 0.001
Sex, no. (%)				< 0.001
Males	120 (31.8)	90 (27.1)	30 (66.7)	
Females	257 (68.2)	242 (72.9)	15 (33.3)	
Smoking status (current or former), no. (%)	66 (17.5)	49 (14.8)	17 (37.8)	< 0.001
Height, cm*	160.10 ± 7.73	159.77 ± 7.61	162.48 ± 8.21	0.027
Weight, kg**	53.00 (47.00–60.00)	53.00 (48.00–60.00)	49.00 (38.95–56.50)	0.004
BMI, kg/m^2^**^a^	20.80 (18.80–22.60)	21.10 (19.30–22.70)	18.2 (15.60–21.10)	< 0.001
Comorbidities, no. (%)				
History of TB	155 (41.1)	131 (39.5)	24 (53.3)	0.076
COPD	33 (8.8)	25 (7.5)	8 (17.8)	0.042
Asthma	29 (7.7)	25 (7.5)	4 (8.9)	0.764
DM	41 (10.9)	34 (10.2)	7 (15.6)	0.305
Cardiovascular disease	44 (11.7)	29 (8.7)	15 (33.3)	< 0.001
Liver disease	20 (5.3)	19 (5.7)	1 (2.2)	0.490
Sputum smear positivity, no. (%)	175 (46.4)	150 (45.2)	25 (55.6)	0.205
CT pattern, no. (%)				< 0.001
FC	42 (11.1)	30 (9.0)	12 (26.7)	
NB with cavity	58 (15.4)	49 (14.8)	9 (20.0)	
NB without cavity	277 (73.5)	253 (76.2)	24 (53.3)	
CT score**	8.00 (6.00–10.00)	8.00 (6.00–10.00)	10.00 (7.00–14.00)	0.003
Cavity, no. (%)				0.002
No cavity	277 (73.5)	253 (76.2)	24 (53.3)	
Cavity	100 (26.5)	79 (23.8)	21 (46.7)	
PNI**	50.95 (47.48–53.50)	51.30 (48.15–54.08)	45.40 (39.11–49.69)	< 0.001
Follow-up duration (days)**	1638.00 (838.00–2587.00)	1767.50 (967.50–2645.25)	792.00 (452.00–1384.00)	< 0.001
Treatment times, no. (%)				0.309
None	199 (52.8)	180 (54.2)	19 (42.2)	
Once	155 (41.1)	132 (39.8)	23 (51.1)	
Twice	23 (6.1)	20 (6.0)	3 (6.6)	
SQF area**	52.98 (34.18–77.69)	55.54 (38.05–79.97)	24.60 (8.79–45.06)	< 0.001
ESM area**	26.93 (22.97–31.42)	26.99 (23.12–31.63)	25.45 (20.53–30.27)	0.096
ESM HU**	36.98 (30.57–43.17)	37.31 (31.78–43.27)	33.18 (24.21–40.98)	0.004

*Mean ± standard deviation.

**Median (IQR).

^a^Seven people without BMI data were excluded from the participants.

BMI, body mass index; TB, tuberculosis; COPD, chronic obstructive pulmonary disease; DM, diabetes mellitus; CT, computed tomography; FC, fibrocavitary; NB, nodular bronchiectatic; PNI, prognostic nutrition index; SQF area, L1 spine level of the cross-sectional area of subcutaneous fat; ESM area, L1 spine level of the cross-sectional area of the erector spinae muscle; ESM HU, L1 spine level of Hounsfield unit of the erector spinae muscle; IQR, interquartile range.

### Correlation between each CT-measured body composition

**Figure 2:**
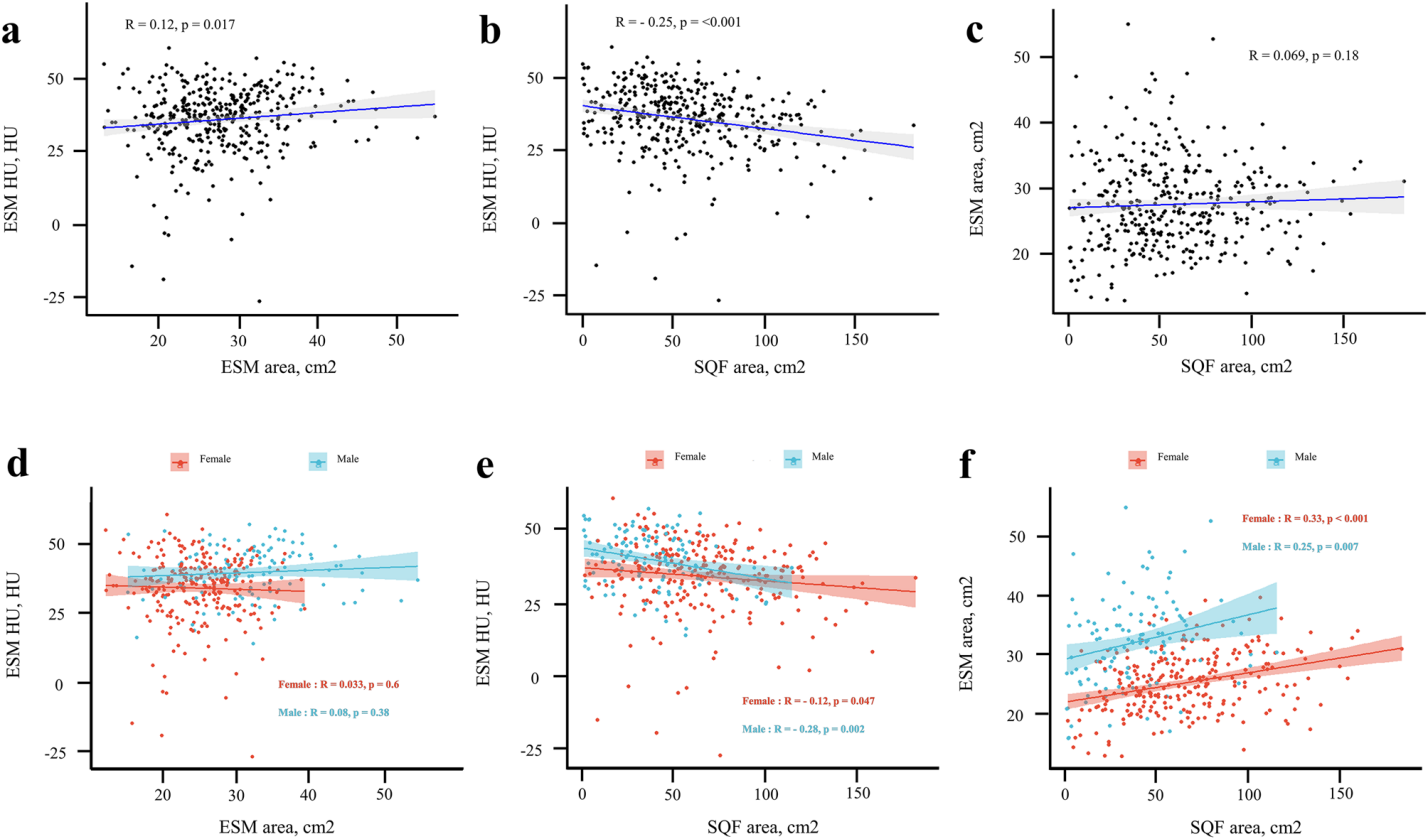
Correlation analysis for each CT-measured body composition (**a**–**c**) and correlation analysis for each CT-measured body composition stratified by sex (**d**–**f**) were represented using scatter plots. ESM HU, L1 spine level of Hounsfield unit of the erector spinae muscle; HU, Hounsfield unit; ESM area, L1 spine level of the cross-sectional area of the erector spinae muscle; SQF area, L1 spine level of the cross-sectional area of subcutaneous fat.

We examined the correlation among the three measured body composition values (ESM area, ESM HU, and SQF area) and observed significant correlations between ESM area and ESM HU and SQF area and ESM HU in total participants, as illustrated in Fig. 2a–c. However, when analyzed by sex, a significant correlation was observed between SQF area and ESM HU, as well as between SQF area and ESM area (Fig. 2d–f).

### Prognostic effect of CT-measured body composition, ESM area, ESM HU, and SQF area on mortality

Table 3 presents the Cox proportional hazard model analysis results evaluating the effect of each body composition value at diagnosis on all-cause mortality of patients with MAC-PD. Regarding the body composition, the SQF area and ESM HU in univariable analysis were associated with decreased mortality of patients with MAC-PD. Multivariable analysis was performed on three models. Model 1 was adjusted for age, sex, and BMI, model 2 was adjusted for sex and considered correlations among continuous variables (Age, BMI, PNI, ESM area, ESM HU, and SQF area) (Supplementary Table S3), and model 3 was adjusted for significant variables in the univariable analysis. In the multivariable analysis, ESM HU continuously had a significantly lower hazard of death. In model 1 and 3, a one-unit increase in ESM HU corresponded to an adjusted hazard ratio (aHR) of 0.95 (95% confidence interval [CI] 0.93–0.97). In model 2, the adjusted hazard ratio associated with a one-unit increase in ESM HU was 0.96 (95% CI 0.94–0.98).

**Table 3:** Cox proportional hazards analysis of all-cause mortality in patients with MAC-PD.

Variable	Univariable analysis		Multivariable analysis (Model 1)		Multivariable analysis (Model 2)		Multivariable analysis (Model 3)	
	HR (95% CI)	*p*-value	HR (95% CI)	*p*-value	HR (95% CI)	*p*-value	HR (95% CI)	*p*-value
Age	1.12 (1.08–1.16)	< 0.001	1.08 (1.03–1.12)	< 0.001	1.07 (1.03–1.12)	0.001	1.07 (1.03–1.12)	0.001
Females	0.23 (0.13–0.43)	< 0.001	0.17 (0.07–0.43)	< 0.001	0.19 (0.08–0.47)	< 0.001	0.17 (0.06–0.48)	0.001
BMI^a^	0.75 (0.67–0.84)	< 0.001	0.79 (0.67–0.93)	0.006	0.80 (0.68–0.94)	0.008	0.80 (0.67–0.94)	0.009
Smoking	2.78 (1.52–5.08)	0.001					1.21 (0.54–2.68)	0.643
TB_Hx	1.41 (0.78–2.53)	0.257						
COPD	2.21 (1.03–4.74)	0.043					1.26 (0.53–3.02)	0.600
Asthma	1.33 (0.47–3.72)	0.590						
DM	1.50 (0.67–3.35)	0.329						
CV	3.44 (1.85–6.40)	< 0.001					1.13 (0.59–2.19)	0.710
LiverDz	0.31 (0.04–2.25)	0.247						
AFB	1.17 (0.65–2.11)	0.609						
CT score	1.17 (1.08–1.26)	< 0.001					1.08 (0.98–1.19)	0.117
PNI	0.89 (0.86–0.92)	< 0.001			0.95 (0.91–0.99)	0.025	0.97 (0.92–1.01)	0.149
SQF area	0.97 (0.95–0.98)	< 0.001	0.99 (0.97–1.01)	0.366	0.99 (0.97–1.01)	0.459	1.00 (0.97–1.02)	0.652
ESM area	0.97 (0.93–1.02)	0.224	0.99 (0.94–1.04)	0.741	0.99 (0.94–1.04)	0.736	0.99 (0.93–1.05)	0.725
ESM HU	0.96 (0.95–0.98)	< 0.001	0.95 (0.93–0.97)	< 0.001	0.96 (0.94–0.98)	< 0.001	0.95 (0.93–0.97)	< 0.001

^a^Seven people without BMI data were excluded from the participants.

Model 1 was adjusted for age, sex, and BMI.

Model 2 was adjusted for sex and considered correlations among continuous variables (Age, BMI, PNI, ESM area, ESM HU, and SQF area) (Supplementary Table 3).

Model 3 was adjusted for statistically significant variables in univariable analysis.

BMI, body mass index; TB Hx, tuberculosis history; COPD, chronic obstructive pulmonary disease; DM, diabetes mellitus; CV, cardiovascular diseases; LiverDz, liver diseases; AFB, acid-fast bacilli; PNI, prognostic nutrition index; SQF area, L1 spine level of the cross-sectional area of subcutaneous fat; ESM area, L1 spine level of the cross-sectional area of the erector spinae muscle; ESM HU, L1 spine level of Hounsfield unit of the erector spinae muscle.

In addition, the effect of ESM HU on the mortality of MAC-PD was consistent in the subgroup analysis according to sex. The aHR values for death in model 3 were 0.93 (95% CI 0.88–0.97, Supplementary Table S4) in males and 0.94 (95% CI 0.91–0.97, Supplementary Table S5) in females.

As body composition differences according to BMI groups were observed (Supplementary Table S6), subgroup analysis according to BMI was also performed. ESM HU had a significantly lower hazard of death (aHR 0.93, 95% CI 0.89–0.97) in the BMI < 18.5 kg/m^2^ group. ESM HU (aHR 0.92, 95% CI 0.88–0.97) and SQF area (aHR 0.98, 95% CI 0.95–1.00) were significant prognostic factors for death in the BMI ≥ 18.5 kg/m^2^ group (Supplementary Tables S7–S8).

### Relation of myosteatosis with mortality

**Figure 3:**
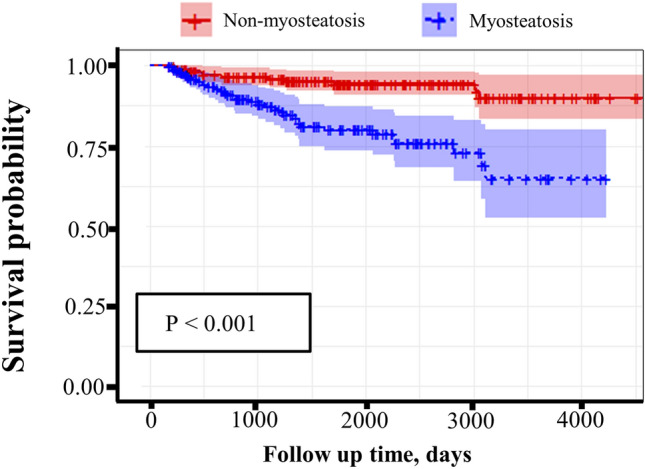
Kaplan–Meier curve stratified by myosteatosis in patients with MAC-PD. ESM HU, L1 spine level of Hounsfield unit of the erector spinae muscle; Myosteatosis, patients with MAC-PD who had < 40.54 HU in males and < 36.06 HU in females; Non-myosteatosis, patients with MAC-PD who had ≥ 40.54 HU in males and ≥ 36.06 HU in females.

ESM HU was associated with MAC-PD mortality; therefore, we classified patients into two groups based on ESM HU: myosteatosis and non-myosteatosis groups. There was a difference in survival between the myosteatosis and non-myosteatosis groups. Patients with MAC-PD and myosteatosis had a lower survival probability than those without myosteatosis (*p* < 0.001, Fig. 3). In addition, the differences in survival rate according to myosteatosis in each subgroup according to sex and BMI were observed (Supplementary Fig. S1).

## Discussion

Few studies have shown the impact of the qualitative CT-measured body composition on the prognosis of patients with MAC-PD, and each finding is conflicting. In our study, myosteatosis—represented by a lower ESM HU—was associated with mortality in patients with MAC-PD independently of the other covariates, including sex and BMI. In addition, subcutaneous fat protected against mortality in patients with MAC-PD and BMI ≥ 18.5 kg/m^2^. There were different effects of fat tissue in skeletal muscles, which was the main factor in lowering the ESM HU^18^, and subcutaneous fat on mortality of patients with MAC-PD in our study.

Previous studies have revealed that obesity has a positive impact on the prognosis of chronic diseases, such as cancer^19^, chronic heart disease, and chronic obstructive pulmonary disease^20,21^. This phenomenon is known as the obesity paradox. However, it has been suggested that not only BMI but also various aspects of body composition such as muscle, fat mass, and myosteatosis have additional prognostic effects^22–24^. In NTM-PD, low BMI is a known risk factor for both its developing^6^ and mortality^7,25^. However, research on the impact of specific body composition, especially myosteatosis, on NTM-PD prognosis remains limited. During the aging process or inflammation-induced lipolysis, the redistribution of fat leads to lipid infiltration into skeletal muscles, resulting in intramyocellular lipids that possess distinct characteristics from subcutaneous fat^12,18^. It enhances the secretion of pro-inflammatory cytokines and can be associated with metabolic disorders, such as insulin resistance and inflammation^12^. Myosteatosis is also associated with reduced muscle strength, compromised physical performance, and decreased physical activity^26–28^. Therefore, early detection of myosteatosis is essential. In our result, myosteatosis was associated with a poor prognosis in MAC-PD, underscoring the importance of investigating myosteatosis in MAC-PD patients to consider early therapeutic interventions.

For the subcutaneous fat, similar to patients with cancer^22^, a protective effect in BMI ≥ 18.5 kg/m^2^ group was observed in our study. Subcutaneous adipose tissue has a higher rate of leptin secretion than other visceral adipose tissue^29^. Leptin is associated with the production of host-protecting cytokines, such as tumor necrosis factor-α and interleukin-12^30^. Therefore, in the BMI ≥ 18.5 kg/m^2^ group with a higher proportion of subcutaneous fat (Supplementary Table S6), the potential secretion of leptin against NTM might be associated with a protective effect on mortality in patients with MAC-PD.

Unlike previous studies^9^, the ESM area did not affect mortality in our study. We measured muscle mass through the ESM area at a specific level. However, even if muscle mass was measured in the same cross-sectional area, actual total body mass and estimated value differences may occur depending on muscle distribution or height. ESM area can be adjusted with height, weight, or BMI. However, it was not considered in our analysis because the variable could be estimated twice during statistical analysis, resulting in overfitting.

This study has several strengths. First, a low ESM HU at diagnosis was associated with increased mortality of patients with MAC-PD. Therefore, myosteatosis could be used to determine treatment initiation for early-stage patients before radiological deterioration. Determining treatment initiation in MAC-PD has always been controversial, and radiological changes, such as cavity development, are suggested markers of treatment initiation^7^. If myosteatosis could predict the mortality of patients with MAC-PD, it may be helpful to classify the group that needs preemptive treatment in advance when diagnosing MAC-PD. Second, our study suggests the possibility of adjuvant non-pharmacological treatment in MAC-PD. As myosteatosis is associated with a poor prognosis in patients with MAC-PD, prognosis improvement can be expected if there is a way to reduce myosteatosis. Previous studies have revealed that higher muscle attenuation is associated with increased muscle strength^26^, and exercise can decrease myosteatosis^31^. Therefore, exercise can be considered as one of non-pharmacological treatment to increase the ESM HU. To verify this, it is necessary to confirm the effect of physical activity and exercise on MAC-PD development or prognosis as a follow-up study.

Despite these strengths, this study has some limitations. First, this was a single-center study, and regional and national differences should be considered in interpreting the results. Second, we measured the body composition at diagnosis, and the CT-measured body composition values may change during follow-up. However, due to the limitations of retrospective studies, there was inadequate follow-up CT information. In future prospective studies, it is necessary to investigate the value of CT-measured body composition in the course of disease progression and whether this value is associated with prognosis. Third, we excluded the participants with malignancy co-morbidities. Given the prevalence of multiple co-morbidities in NTM-PD^32^, further analysis is warranted encompassing patients with diverse co-morbidities. Finally, when determining myosteatosis, the median value of ESM HU was considered as the cut-off in the patient group. However, to determine a statistically accurate cut-off value, comparing the ESM HU between the control and deceased groups is necessary. Therefore, it is necessary to identify the cut-off value that will be the standard ESM HU in future studies.

In conclusion, our study revealed that a low ESM HU, which indirectly reflects myosteatosis, was associated with increased mortality in patients with MAC-PD. Therefore, it is necessary to consider the impact of qualitative body composition as well as quantitative body composition when evaluating MAC-PD prognosis.

## Methods

### Ethical standards

The study protocol was reviewed and approved by the Institutional Review Board of the Severance Hospital Ethics Committee (IRB Approval Number: 4–2022-0523). Informed consent was waived by the Institutional Review Board of the Severance Hospital Ethics Committee. All methods were performed in accordance with the Declaration of Helsinki.

### Study population

Patients with MAC-PD diagnosed based on 2007 American Thoracic Society and Infectious Disease Society of America guidelines were eligible for this study^13^. We excluded patients with co-morbidities, such as transplantation and malignancies, which have the potential to significantly influence mortality, those with a short follow-up duration (< 6 months), those without a CT image at diagnosis, and those with inappropriate CT scans for measure body composition. Inappropriate CT included motion artifacts within the images, a slice thickness exceeding 5 mm, inadequate inclusion of the L1 level, absence of available soft kernel-reconstructed images, and a lack of available noncontrast CT images^33^.

### Data collection and definition

Data on clinico-demographic factors, including age, sex, height, weight, BMI, smoking history, medical history, AFB smear and culture, laboratory findings and chest CT findings at the time of diagnosis, and anti-MAC treatment history were collected. The primary clinical outcome was all-cause mortality during the follow-up. The death outcome was confirmed by collecting medical records and referring to Statistics Korea for data on the causes of death between 2005 and 2018. Myosteatosis is fat infiltration into the skeletal muscles and was defined as the ESM HU value. Less than the median value of ESM HU led to classification as myosteatosis and more than the median value of ESM HU to classification as non-myosteatosis. Owing to the difference in ESM HU distribution between males and females, the median of ESM HU criterion was applied differently according to sex.

### Radiologic measurement

**Figure 4:**
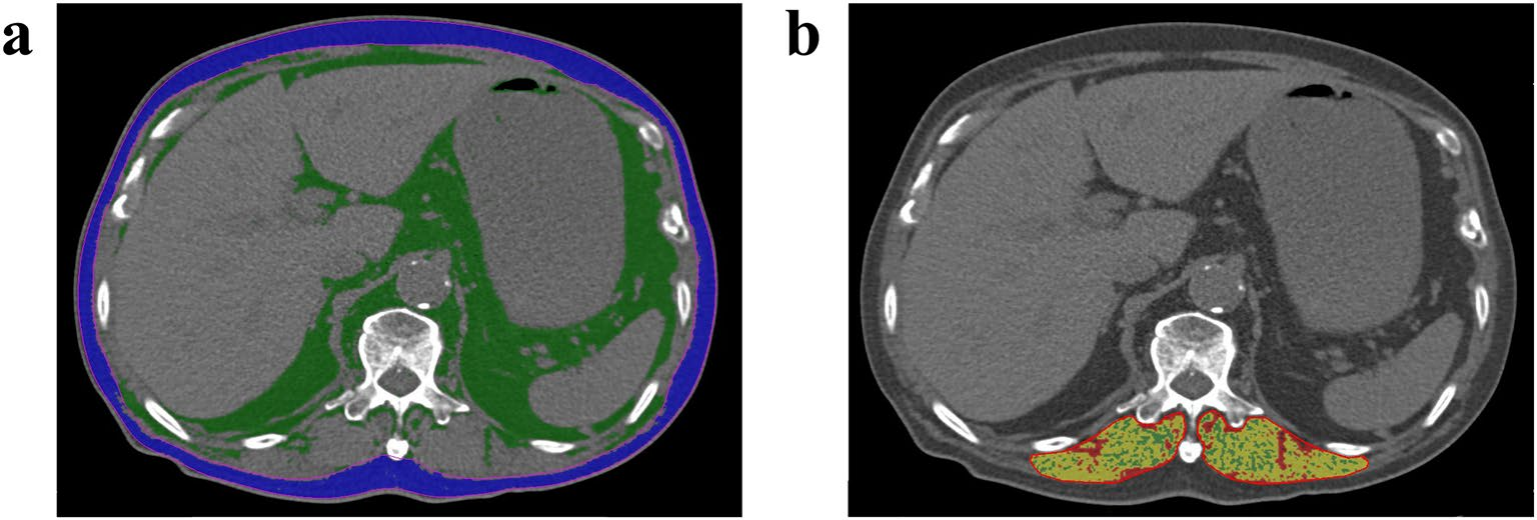
Computed tomography images show how body compositions are measured. (**a**) An image measuring the cross-sectional area of subcutaneous fat at L1 spine level (blue area). (**b**) An image measuring the cross-sectional area and Hounsfield unit of erector spinae muscles at L1 spine level (yellow area surrounded by red lines indicates muscle with a threshold of − 29 to + 150 HU; red and green areas indicate intermuscular fat with − 200 to − 100 HU and − 100 to − 30 HU, respectively).

Body composition was measured using non-contrast chest CT before and after 3 months of diagnosis with MAC-PD. Chest CT scans were obtained from the level of the supraclavicular fossae to the adrenal glands under an inspiratory breath-hold with the following scanning parameters: tube voltage of 120 kVp, reference tube current of 100 mAs, average pitch of 1, and volume CT dose index < 7.0 mGy. After scanning, axial images were reconstructed using a slice 1 mm thick and a slice increment of 1 mm with a medium-smooth convolution kernel. Thereafter, CT images were transferred to a software system (Aquarius iNtuition; Version 4.4.11, TeraRecon, Durham, North Carolina, The United States of America) for analysis. The program was used to measure three body compositions: ESM area, ESM HU, and SQF area. After reconstructing the CT images at 3 mm slice thickness and intervals, a single evaluator measured body compositions at the middle of the L1 vertebra. For the erector spinae muscle, manual segmentation was done using a region of interest drawn along the muscle outline avoiding the edges to reduce partial volume averaging from the muscle-fat interface. Subsequently, based on the area histogram, pixels with the predefined threshold of − 29 to + 150 HU were incorporated for the analysis of the ESM. Semi-automatic segmentation was performed for the subcutaneous fat with a threshold of − 190 to − 30 HU (Fig. 4).

Another evaluator measured the CT score, which is the variable related to the severity of NTM-PD, according to a prior published scoring system^34^. A total CT score of 30 was allocated to determine each patient's overall extent of lung lesions. Both readers were blinded to the clinical information, including medical history and outcomes.

### Statistical analysis

Categorical variables are presented as numbers and percentages. Continuous variables with normal distribution are presented as mean and standard deviation, and those with non-normal distribution are presented as median (interquartile range [IQR]). The Chi-square and Fisher’s exact tests were used to compare categorical variables. In contrast, the Student’s *t*-test and Mann–Whitney U-test were used to compare continuous variables. We conducted Cox proportional hazard regression analysis to assess the hazard ratio of body composition in relation to mortality of patients with MAC-PD. The log-rank test was conducted to compare the mortality between the myosteatosis and non-myosteatosis groups. A two-tailed statistical significance was defined as a *p*-value < 0.05. SPSS (Version 26.0, IBM corporation, Armonk, New York, The United States of America) and R software (version × 64 4.2.1, R Foundation for Statistical Computing, Vienna, Austria) were used to perform all statistical analyses.

### Supplementary Information

Supplementary Information.

## Data Availability

The datasets generated during and/or analyzed during the current study are available from the corresponding author on reasonable request.
