# Supplementary material for: Myosteatosis as a prognostic factor of Mycobacterium avium complex pulmonary disease
Source: Sci Rep. 2023 Aug 22;13:13680. doi: 10.1038/s41598-023-40984-y (PMC10444847; doi:10.1038/s41598-023-40984-y)

**SUPPLEMENTARY INFORMATION**

**Myosteatosis as a prognostic factor of *Mycobacterium avium* complex pulmonary disease**

Eunki Chung^a^, Youngmok Park^a^, Song Yee Kim^a^, Moo Suk Park^a^, Young Sam Kim^a^, Hye-Jeong Lee^b^*, Young Ae Kang^a^*

^a^Division of Pulmonary and Critical Care Medicine, Department of Internal Medicine, Severance Hospital, Institute for Immunology and Immunological Disease, Yonsei University College of Medicine, Seoul, Republic of Korea

^b^Department of Radiology, Research Institute of Radiological Science, Severance Hospital, Yonsei University College of Medicine, Seoul, Republic of Korea

* Hye-Jeong Lee and Young Ae Kang contributed equally to this paper.

**Supplementary Table S1.** Baseline characteristics of male participants with *Mycobacterium avium* complex pulmonary disease (MAC-PD)

|  | **Total participants**  **(n = 120)** | **Survivors**  **(n = 90)** | **Deaths**  **(n = 30)** | **p-value** |
| --- | --- | --- | --- | --- |
| **Characteristics** |  |  |  |  |
| **Age (years)*** | 68.60 ± 10.06 | 67.44 ± 10.35 | 72.07 ± 8.35 | 0.029 |
| **Smoking status (Current or former), no. (%)** | 63 (52.5) | 46 (51.1) | 17 (56.7) | 0.675 |
| **Height, cm*** | 167.40 ± 5.84 | 167.48 ± 6.17 | 167.19 ± 4.85 | 0.818 |
| **Weight, kg**** | 58.78 ± 10.06 | 61.07 ± 8.78 | 51.97 ± 10.67 | <0.001 |
| **BMI, kg/m^2^**** ^a^ | 21.40  (18.40–22.70) | 21.90  (20.35–23.05) | 18.15  (15.60–21.00) | <0.001 |
| **Comorbidities, no. (%)** |  |  |  |  |
| History of TB | 59 (49.2) | 42 (46.7) | 17 (56.7) | 0.402 |
| COPD | 22 (18.3) | 16 (17.8) | 6 (20.0) | 0.789 |
| Asthma | 7 (5.8) | 6 (6.7) | 1 (3.3) | 0.679 |
| DM | 17 (14.2) | 11 (12.2) | 6 (20.0) | 0.364 |
| Cardiovascular disease | 29 (24.2) | 18 (20.0) | 11 (36.7) | 0.085 |
| Liver disease | 9 (7.5) | 8 (8.9) | 1 (3.3) | 0.447 |
| **Sputum smear positivity,**  **no. (%)** | 59 (49.2) | 39 (43.3) | 20 (66.7) | 0.035 |
| **CT pattern, no. (%)** |  |  |  | 0.004 |
| FC | 21 (17.5) | 11 (12.2) | 10 (33.3) |  |
| NB with cavity | 11 (9.2) | 6 (6.7) | 5 (16.7) |  |
| NB without cavity | 88 (73.3) | 73 (81.1) | 15 (50.0) |  |
| **CT score**** | 8.00 (6.00–10.00) | 8.00 (6.00–10.00) | 9.00 (6.00–12.25) | 0.136 |
| **Cavity, no. (%)** |  |  |  | 0.002 |
| No cavity | 88 (73.3) | 73 (81.1) | 15 (50.0) |  |
| Cavity | 32 (26.7) | 17 (18.9) | 15 (50.0) |  |
| **PNI**** | 48.33 ± 6.11 | 49.73 ± 5.43 | 44.19 ± 6.20 | <0.001 |
| **Follow-up duration (days)**** | 1719.50  (749.25–2666.50) | 1918.50  (1201.50–2756.50) | 749.50  (383.00–1379.25) | <0.001 |
| **Treatment times, no. (%)** |  |  |  | 0.044 |
| None | 58 (48.3) | 49 (54.4) | 9 (30.0) |  |
| Once | 57 (47.5) | 38 (42.2) | 19 (63.3) |  |
| Twice | 5 (4.2) | 3 (3.3) | 2 (6.7) |  |
| **SQF area, cm^2^**** | 36.79  (21.13–53.77) | 42.11  (24.97–55.51) | 23.61  (7.85–39.05) | 0.002 |
| **ESM area, cm^2^**** | 32.54  (27.03–36.19) | 33.55  (29.53–36.91) | 27.41  (24.36–32.50) | <0.001 |
| **ESM HU**** | 39.75 ± 9.02 | 40.61 ± 8.54 | 37.19 ± 10.04 | 0.072 |

*Mean ± standard deviation

** Median (IQR)

^a^ Seven people without BMI data were excluded from the participants.

BMI, body mass index; TB, tuberculosis; COPD, chronic obstructive pulmonary disease; DM, diabetes mellitus; CT, computed tomography; FC, fibrocavitary; NB, nodular bronchiectatic; PNI, prognostic nutrition index; SQF area, L1 spine level of the cross-sectional area of subcutaneous fat; ESM area, L1 spine level of the cross-sectional area of the erector spinae muscle; ESM HU, L1 spine level of Hounsfield unit of the erector spinae muscle; IQR, interquartile range.

**Supplementary Table S2.** Baseline characteristics of female participants with MAC-PD

|  | **Total participants**  **(n = 257)** | **Survivors**  **(n = 242)** | **Deaths**  **(n = 15)** | **p-value** |
| --- | --- | --- | --- | --- |
| **Characteristics** |  |  |  |  |
| **Age (years)*** | 61.68 ± 11.34 | 60.66 ± 10.61 | 78.07 ± 10.53 | <0.001 |
| **Smoking status (Current or former), no. (%)** | 3 (1.2) | 3 (1.2) | 0 (0.0) | 1.000 |
| **Height, cm*** | 156.64 ± 5.88 | 156.87 ± 5.89 | 153.07 ± 4.54 | 0.015 |
| **Weight, kg**** | 52.00  (46.00–56.00) | 52.00  (46.43–56.00) | 39.50  (35.46–53.00) | 0.012 |
| **BMI, kg/m^2^**** ^a^ | 20.60  (18.90–22.50) | 20.70  (19.00–22.56) | 18.70  (15.60–21.90) | 0.059 |
| **Comorbidities, no. (%)** |  |  |  |  |
| History of TB | 96 (37.4) | 89 (36.8) | 7 (46.7) | 0.583 |
| COPD | 11 (4.3) | 9 (3.7) | 2 (13.3) | 0.129 |
| Asthma | 22 (8.6) | 19 (7.9) | 3 (20.0) | 0.126 |
| DM | 24 (9.3) | 23 (9.5) | 1 (6.7) | 1.000 |
| Cardiovascular disease | 15 (5.8) | 11 (4.5) | 4 (26.7) | 0.007 |
| Liver disease | 11 (4.3) | 11 (4.5) | 0 (0.0) | 1.000 |
| **Sputum smear positivity,**  **no. (%)** | 116 (45.1) | 111 (45.9) | 5 (33.3) | 0.428 |
| **CT pattern, no. (%)** |  |  |  | 0.330 |
| FC | 21 (8.2) | 19 (7.9) | 2 (13.3) |  |
| NB with cavity | 47 (18.3) | 43 (17.8) | 4 (26.7) |  |
| NB without cavity | 189 (73.5) | 180 (74.4) | 9 (60.0) |  |
| **CT score**** | 8.00 (6.00–10.00) | 8.00 (6.00–10.00) | 10.00 (7.00–15.00) | 0.004 |
| **Cavity, no. (%)** |  |  |  | 0.234 |
| No cavity | 189 (73.5) | 180 (74.4) | 9 (60.0) |  |
| Cavity | 68 (26.5) | 62 (25.6) | 6 (40.0) |  |
| **PNI**** | 51.53  (48.39–54.55) | 51.85  (48.65–54.85) | 44.85  (40.50–52.90) | 0.003 |
| **Follow-up duration (days)**** | 1567.00  (851.00–2570.50) | 1656.00  (896.25–2590.75) | 1007.00  (472.00–1622.00) | 0.032 |
| **Treatment times, no. (%)** |  |  |  | 0.579 |
| None | 141 (54.9) | 131 (54.1) | 10 (66.7) |  |
| Once | 98 (38.1) | 94 (38.8) | 4 (26.7) |  |
| Twice | 18 (7.0) | 17 (7.0) | 1 (6.7) |  |
| **SQF area, cm^2^**** | 63.94  (41.45–86.30) | 65.11  (43.23–89.46) | 25.82  (18.39–70.48) | <0.001 |
| **ESM area, cm^2^**** | 25.38 ± 5.02 | 25.60 ± 4.93 | 21.93 ± 5.36 | 0.006 |
| **ESM HU**** | 36.06  (28.72–41.36) | 36.65  (30.26–41.54) | 20.16  (3.04–29.02) | <0.001 |

*Mean ± standard deviation

** Median (IQR)

^a^ Seven people without BMI data were excluded from the participants.

BMI, body mass index; TB, tuberculosis; COPD, chronic obstructive pulmonary disease; DM, diabetes mellitus; CT, computed tomography; FC, fibrocavitary; NB, nodular bronchiectatic; PNI, prognostic nutrition index; SQF area, L1 spine level of the cross-sectional area of subcutaneous fat; ESM area, L1 spine level of the cross-sectional area of the erector spinae muscle; ESM HU, L1 spine level of Hounsfield unit of the erector spinae muscle; IQR, interquartile range.

**Supplementary Table S3.** Correlation analysis of continuous variables among total, male, and female participants.

| **Total participants** | | | | | | |
| --- | --- | --- | --- | --- | --- | --- |
| Age | 1.00 |  |  |  |  |  |
| BMI | 0.049 | 1.00 |  |  |  |  |
| ESM area | -0.005 | 0.337* | 1.00 |  |  |  |
| ESM HU | -0.436* | -0.154* | 0.123 | 1.00 |  |  |
| PNI | -0.336* | 0.159* | 0.049 | 0.104 | 1.00 |  |
| SQF area | -0.035 | 0.642* | 0.069 | -0.255* | 0.221* | 1.00 |
|  | Age | BMI | ESM area | ESM HU | PNI | SQF area |
| **Male participants** | | | | | | |
| Age | 1.00 |  |  |  |  |  |
| BMI | -0.126 | 1.00 |  |  |  |  |
| ESM area | -0.403* | 0.365* | 1.00 |  |  |  |
| ESM HU | -0.491* | -0.118 | 0.109 | 1.00 |  |  |
| PNI | -0.294* | 0.239 | 0.392* | 0.176 | 1.00 |  |
| SQF area | 0.023 | 0.694* | 0.287* | -0.242* | 0.160 | 1.00 |
|  | Age | BMI | ESM area | ESM HU | PNI | SQF area |
| **Female participants** | | | | | | |
| Age | 1.00 |  |  |  |  |  |
| BMI | 0.128 | 1.00 |  |  |  |  |
| ESM area | -0.043 | 0.328* | 1.00 |  |  |  |
| ESM HU | -0.560* | -0.196* | -0.029 | 1.00 |  |  |
| PNI | -0.231* | 0.131 | 0.090 | 0.150 | 1.00 |  |
| SQF area | 0.145 | 0.752* | 0.320* | -0.151 | 0.144 | 1.00 |
|  | Age | BMI | ESM area | ESM HU | PNI | SQF area |

* The correlation is significant at the 0.05 level.

**Supplementary Table S4.** Cox proportional hazards analyses of all-cause mortality in males with MAC-PD

|  | **Univariable analysis** | | **Multivariable analysis**  **(Model 1)** | | **Multivariable analysis**  **(Model 2)** | | **Multivariable analysis**  **(Model 3)** | |
| --- | --- | --- | --- | --- | --- | --- | --- | --- |
| **Variable** | **HR (95% CI)** | **p-value** | **HR (95% CI)** | **p-value** | **HR (95% CI)** | **p-value** | **HR (95% CI)** | **p-value** |
| Age | 1.06 (1.02–1.10) | 0.007 | 1.02 (0.97–1.07) | 0.508 | 1.02 (0.97–1.08) | 0.406 | 1.03 (0.98–1.09) | 0.272 |
| BMI ^a^ | 0.72 (0.62–0.82) | <0.001 | 0.69 (0.56–0.84) | <0.001 | 0.75 (0.61–0.92) | 0.006 | 0.74 (0.59–0.92) | 0.006 |
| Smoking | 1.09 (0.53–2.25) | 0.812 |  |  |  |  |  |  |
| TB_Hx | 1.26 (0.61–2.60) | 0.537 |  |  |  |  |  |  |
| COPD | 1.17 (0.47–2.87) | 0.738 |  |  |  |  |  |  |
| Asthma | 0.47 (0.06–3.44) | 0.455 |  |  |  |  |  |  |
| DM | 1.44 (0.59–3.52) | 0.427 |  |  |  |  |  |  |
| CV | 1.76 (0.84–3.70) | 0.136 |  |  |  |  |  |  |
| LiverDz | 0.33 (0.04–2.43) | 0.277 |  |  |  |  |  |  |
| AFB | 2.02 (0.94–4.33) | 0.070 |  |  |  |  |  |  |
| CT score | 1.15 (1.03–1.28) | 0.014 |  |  |  |  | 1.08 (0.95–1.22) | 0.246 |
| PNI | 0.86 (0.81–0.91) | <0.001 |  |  | 0.92 (0.86–0.99) | 0.017 | 0.93 (0.86–0.99) | 0.033 |
| SQF area | 0.98 (0.96–0.99) | 0.012 | 1.01 (0.98–1.03) | 0.581 | 1.01 (0.98–1.03) | 0.695 | 1.01 (0.98–1.04) | 0.581 |
| ESM area | 0.92 (0.87–0.97) | 0.002 | 0.99 (0.93–1.04) | 0.640 | 1.00 (0.94–1.06) | 0.983 | 1.01 (0.95–1.07) | 0.801 |
| ESM HU | 0.94 (0.90–0.97) | 0.002 | 0.93 (0.88–0.98) | 0.003 | 0.93 (0.88–0.97) | 0.002 | 0.93 (0.88–0.97) | 0.002 |

^a^ Seven people without BMI data were excluded from the participants.

Model 1 was adjusted for age and BMI.

Model 2 was adjusted for considered correlations among continuous variables (Age, BMI, PNI, ESM area, ESM HU, and SQF area) (Supplementary Table 3)

Model 3 was adjusted for statistically significant variables in univariable analysis.

BMI, body mass index; TB Hx, tuberculosis history; COPD, chronic obstructive pulmonary disease; DM, diabetes mellitus; CV, cardiovascular diseases; LiverDz, liver diseases; AFB, acid-fast bacilli; PNI, prognostic nutrition index; SQF area, L1 spine level of the cross-sectional area of subcutaneous fat; ESM area, L1 spine level of the cross-sectional area of the erector spinae muscle; ESM HU, L1 spine level of Hounsfield unit of the erector spinae muscle.

**Supplementary Table S5.** Cox proportional hazards analyses of all-cause mortality in females with MAC-PD

|  | **Univariable analysis** | | **Multivariable analysis**  **(Model 1)** | | **Multivariable analysis**  **(Model 2)** | | **Multivariable analysis**  **(Model 3)** | |
| --- | --- | --- | --- | --- | --- | --- | --- | --- |
| **Variable** | **HR (95% CI)** | **p-value** | **HR (95% CI)** | **p-value** | **HR (95% CI)** | **p-value** | **HR (95% CI)** | **p-value** |
| Age | 1.21 (1.13–1.29) | <0.001 | 1.16 (1.08-1.25) | <0.001 | 1.16 (1.08–1.25) | <0.001 | 1.20 (1.11–1.31) | <0.001 |
| BMI ^a^ | 0.77 (0.64–0.93) | 0.007 | 1.04 (0.79-1.35) | 0.795 | 1.04 (0.79–1.35) | 0.798 | 1.03 (0.77–1.38) | 0.834 |
| Smoking | 0.05 (0.00–inf) | 0.814 |  |  |  |  |  |  |
| TB_Hx | 1.19 (0.43–3.30) | 0.736 |  |  |  |  |  |  |
| COPD | 2.83 (0.64–12.61) | 0.172 |  |  |  |  |  |  |
| Asthma | 3.66 (1.01–13.33) | 0.049 |  |  |  |  | 2.17 (0.33–14.47) | 0.423 |
| DM | 0.68 (0.09–5.21) | 0.714 |  |  |  |  |  |  |
| CV | 4.72 (1.50–14.88) | 0.008 |  |  |  |  | 0.29 (0.05–1.88) | 0.196 |
| LiverDz | 0.05 (0.00–760.91) | 0.533 |  |  |  |  |  |  |
| AFB | 0.40 (0.13–1.21) | 0.106 |  |  |  |  |  |  |
| CT score | 1.24 (1.09–1.42) | 0.001 |  |  |  |  | 1.09 (0.92–1.28) | 0.326 |
| PNI | 0.90 (0.85–0.95) | <0.001 |  |  | 1.00 (0.93–1.08) | 0.948 | 1.04 (0.96–1.13) | 0.316 |
| SQF area | 0.97 (0.95–0.99) | 0.003 | 0.97 (0.94–1.00) | 0.062 | 0.97 (0.94–1.00) | 0.062 | 0.98 (0.94–1.01) | 0.146 |
| ESM area | 0.88 (0.79–0.98) | 0.018 | 0.97 (0.84–1.12) | 0.666 | 0.97 (0.84–1.13) | 0.687 | 0.90 (0.76–1.08) | 0.253 |
| ESM HU | 0.94 (0.92–0.96) | <0.001 | 0.95 (0.93–0.98) | 0.001 | 0.95 (0.93–0.98) | 0.001 | 0.94 (0.91–0.97) | <0.001 |

^a^ Seven people without BMI data were excluded from the participants.

Model 1 was adjusted for age and BMI.

Model 2 was adjusted for considered correlations among continuous variables (Age, BMI, PNI, ESM area, ESM HU, and SQF area) (Supplementary Table 3)

Model 3 was adjusted for statistically significant variables in univariable analysis.

BMI, body mass index; TB Hx, tuberculosis history; COPD, chronic obstructive pulmonary disease; DM, diabetes mellitus; CV, cardiovascular diseases; LiverDz, liver diseases; AFB, acid-fast bacilli; PNI, prognostic nutrition index; SQF area, L1 spine level of the cross-sectional area of subcutaneous fat; ESM area, L1 spine level of the cross-sectional area of the erector spinae muscle; ESM HU, L1 spine level of Hounsfield unit of the erector spinae muscle.

**Supplementary Table S6.** Computed tomography (CT)-measured body composition of participants with MAC-PD classified according to BMI

|  | **Total participants**  **(n = 370)^a^** | **BMI < 18.5 kg/m^2^**  **(n = 81)** | **BMI ≥ 18.5 kg/m^2^**  **(n = 289)** | **p-value** |
| --- | --- | --- | --- | --- |
| **Variable** |  |  |  |  |
| SQF area, cm^2^** | 52.98 (34.18–77.69) | 22.22 (10.58–36.03) | 62.52 (45.42–84.77) | <0.001 |
| ESM area, cm^2^** | 26.93 (22.97–31.42) | 23.77 (20.74–27.76) | 27.96 (23.71–32.47) | <0.001 |
| ESM HU** | 36.98 (30.57–43.17) | 37.07 (32.61–42.47) | 37.06 (29.90–43.27) | 0.563 |

** Median (IQR)

^a^ Seven people without BMI data were excluded from the participants.

SQF area, L1 spine level of the cross-sectional area of subcutaneous fat; ESM area, L1 spine level of the cross-sectional area of the erector spinae muscle; ESM HU, L1 spine level of Hounsfield unit of the erector spinae muscle; IQR, interquartile range.

**Supplementary Table S7.** Cox proportional hazards analyses of all-cause mortality of patients with MAC-PD in BMI < 18.5 kg/m^2^ group

|  | **Univariable analysis** | | **Multivariable analysis**  **(Model 1)** | | | **Multivariable analysis**  **(Model 2)** | | **Multivariable analysis**  **(Model 3)** | | |
| --- | --- | --- | --- | --- | --- | --- | --- | --- | --- | --- |
| **Variable** | **HR (95% CI)** | **p-value** | **HR (95% CI)** | **p-value** | | **HR (95% CI)** | **p-value** | **HR (95% CI)** | **p-value** | |
| Age | 1.09 (1.04–1.13) | <0.001 | 1.05 (0.99–1.11) | | 0.119 | 1.05 (0.98–1.12) | 0.148 | 1.06 (1.00–1.13) | | 0.043 |
| Females | 0.19 (0.08–0.47) | <0.001 | 0.19 (0.06–0.63) | | 0.007 | 0.20 (0.06–0.64) | 0.006 | 0.10 (0.03–0.37) | | 0.001 |
| Smoking | 2.88 (1.27–6.53) | 0.011 |  | |  |  |  | 0.96 (0.36–2.57) | | 0.942 |
| TB_Hx | 1.01 (0.46–2.22) | 0.979 |  | |  |  |  |  | |  |
| COPD | 2.17 (0.90–5.21) | 0.084 |  | |  |  |  |  | |  |
| Asthma | 1.38 (0.19–10.30) | 0.752 |  | |  |  |  |  | |  |
| DM | 2.90 (0.98–8.59) | 0.055 |  | |  |  |  |  | |  |
| CV | 1.70 (0.68–4.26) | 0.259 |  | |  |  |  |  | |  |
| LiverDz | 0.05 (0–1153.43) | 0.553 |  | |  |  |  |  | |  |
| AFB | 1.21 (0.54–2.75) | 0.644 |  | |  |  |  |  | |  |
| CT score | 1.17 (1.06–1.30) | 0.001 |  | |  |  |  | 1.25 (1.11–1.40) | | <0.001 |
| PNI | 0.90 (0.84–0.96) | 0.001 |  | |  | 0.91 (0.84–0.98) | 0.014 | 0.94 (0.87–1.02) | | 0.114 |
| SQF area | 0.96 (0.94–0.99) | 0.017 | 0.97 (0.94–1.01) | | 0.131 | 0.98 (0.95–1.02) | 0.377 | 0.98 (0.94–1.02) | | 0.372 |
| ESM area | 1.03 (0.97–1.09) | 0.324 | 1.02 (0.95–1.09) | | 0.617 | 1.03 (0.97–1.11) | 0.331 | 1.05 (0.98–1.12) | | 0.184 |
| ESM HU | 0.96 (0.94–0.99) | 0.004 | 0.95 (0.91–0.98) | | 0.001 | 0.95 (0.91–0.98) | 0.003 | 0.93 (0.89–0.97) | | <0.001 |

Model 1 was adjusted for age and sex.

Model 2 was adjusted for sex and considered correlations among continuous variables (Age, PNI, ESM area, ESM HU, and SQF area) (Supplementary Table 3)

Model 3 was adjusted for statistically significant variables in the univariable analysis.

BMI, body mass index; TB Hx, tuberculosis history; COPD, chronic obstructive pulmonary disease; DM, diabetes mellitus; CV, cardiovascular diseases; LiverDz, liver diseases; AFB, acid-fast bacilli; PNI, prognostic nutrition index; SQF area, L1 spine level of the cross-sectional area of subcutaneous fat; ESM area, L1 spine level of the cross-sectional area of the erector spinae muscle; ESM HU, L1 spine level of Hounsfield unit of the erector spinae muscle.

**Supplementary Table S8.** Cox proportional hazards analyses of all-cause mortality of patients with MAC-PD in BMI ≥ 18.5 kg/m^2^ group

|  | **Univariable analysis** | | **Multivariable analysis**  **(Model 1)** | | | **Multivariable analysis**  **(Model 2)** | | **Multivariable analysis**  **(Model 3)** | |
| --- | --- | --- | --- | --- | --- | --- | --- | --- | --- |
| **Variable** | **HR (95% CI)** | **p-value** | **HR (95% CI)** | **p-value** | | **HR (95% CI)** | **p-value** | **HR (95% CI)** | **p-value** |
| Age | 1.15 (1.09–1.21) | <0.001 | 1.12 (1.05–1.19) | | <0.001 | 1.12 (1.05–1.20) | 0.001 | 1.12 (1.04–1.20) | 0.002 |
| Females | 0.31 (0.13–0.75) | 0.010 | 0.34 (0.10–1.20) | | 0.093 | 0.33 (0.09–1.21) | 0.096 | 0.47 (0.10–2.27) | 0.348 |
| Smoking | 2.94 (1.20–7.20) | 0.018 |  | |  |  |  | 3.01 (0.66–13.70) | 0.154 |
| TB_Hx | 1.57 (0.65–3.81) | 0.318 |  | |  |  |  |  |  |
| COPD | 0.69 (0.09–5.14) | 0.714 |  | |  |  |  |  |  |
| Asthma | 2.13 (0.62–7.33) | 0.232 |  | |  |  |  |  |  |
| DM | 1.23 (0.36–4.22) | 0.738 |  | |  |  |  |  |  |
| CV | 5.83 (2.41–1414) | <0.001 |  | |  |  |  | 2.68 (0.99–7.29) | 0.053 |
| LiverDz | 0.61 (0.08–4.60) | 0.635 |  | |  |  |  |  |  |
| AFB | 0.77 (0.32–1.90) | 0.576 |  | |  |  |  |  |  |
| CT score | 0.92 (0.78–1.09) | 0.333 |  | |  |  |  |  |  |
| PNI | 0.89 (0.84–0.93) | <0.001 |  | |  | 0.98 (0.91–1.05) | 0.612 | 1.03 (0.95–1.11) | 0.541 |
| SQF area | 0.98 (0.96–1.00) | 0.037 | 0.98 (0.95–1.00) | | 0.029 | 0.98 (0.96–1.00) | 0.054 | 0.98 (0.95–1.00) | 0.045 |
| ESM area | 0.96 (0.90–1.03) | 0.300 | 0.95 (0.88–1.02) | | 0.126 | 0.94 (0.87–1.01) | 0.087 | 0.93 (0.86–1.01) | 0.080 |
| ESM HU | 0.95 (0.93–0.97) | <0.001 | 0.94 (0.92–0.97) | | <0.001 | 0.95 (0.91–0.99) | 0.007 | 0.92 (0.88–0.97) | 0.001 |

Model 1 was adjusted for age and sex.

Model 2 was adjusted for sex and considered correlations among continuous variables (Age, PNI, ESM area, ESM HU, and SQF area) (Supplementary Table 3)

Model 3 was adjusted for statistically significant variables in the univariable analysis.

BMI, body mass index; TB Hx, tuberculosis history; COPD, chronic obstructive pulmonary disease; DM, diabetes mellitus; CV, cardiovascular diseases; LiverDz, liver diseases; AFB, acid-fast bacilli; PNI, prognostic nutrition index; SQF area, L1 spine level of the cross-sectional area of subcutaneous fat; ESM area, L1 spine level of the cross-sectional area of the erector spinae muscle; ESM HU, L1 spine level of Hounsfield unit of the erector spinae muscle.

**Supplementary Figure S1.** Kaplan–Meier curve stratified using ESM HU in patients with MAC-PD according to sex and BMI.

(a) Males with MAC-PD

(b) Females with MAC-PD

(c) BMI < 18.5 kg/m^2^ group of patients with MAC-PD

(d) BMI ≥ 18.5 kg/m^2^ group of patients with MAC-PD

ESM HU, L1 spine level of Hounsfield unit of the erector spinae muscle; Myosteatosis, patients with MAC-PD who had <40.54 HU in males and <36.06 HU in females; Non-myosteatosis, patients with MAC-PD who had ≥40.54 HU in males and ≥36.06 HU in females.


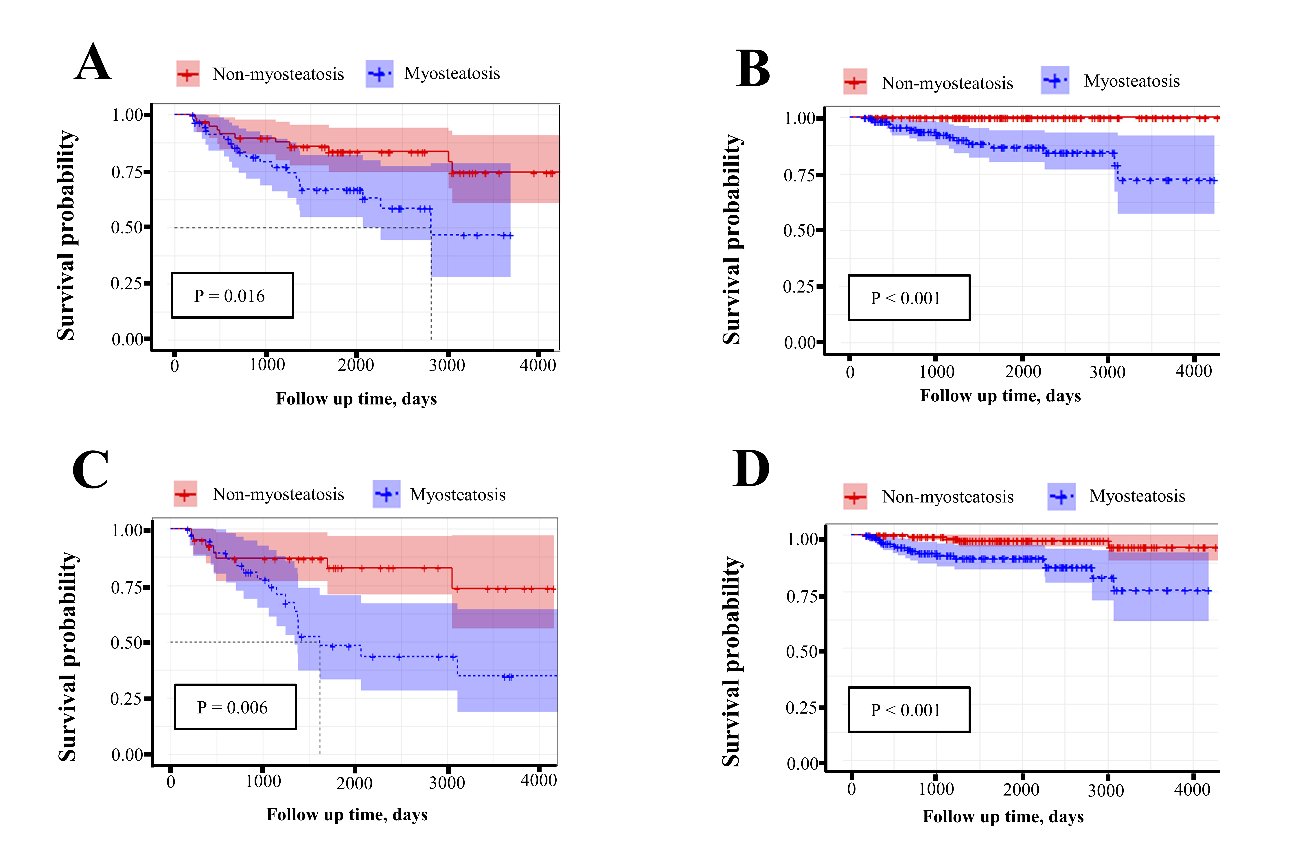

Supplement: Supplementary file 1 — Supplementary Information. [file 41598_2023_40984_MOESM1_ESM.docx]
